# Supplementary material for: Hot spot prediction in protein-protein interactions by an ensemble system
Source: BMC Syst Biol. 2018 Dec 31;12(Suppl 9):132. doi: 10.1186/s12918-018-0665-8 (PMC6311905; doi:10.1186/s12918-018-0665-8)
Supplement: Supplementary file 2 — All relevant MATLAB code. A simple Matlab implement of our predictor. (ZIP 19,742 kb) [file 12918_2018_665_MOESM2_ESM.zip › Additional file 2/Read ASA/ASA Property/SKEMPI/1DQJA.txt]

 NetSurfP - Protein Surface Accessibility and Secondary Structure Predictions

  

|  |  |  |
| --- | --- | --- |
|  |  | NetSurfP - Protein Surface Accessibility and Secondary Structure Predictions  Technical University of Denmark |

  


---

```
# For publication of results, please cite:
# A generic method for assignment of reliability scores applied to solvent accessibility predictions.
# Bent Petersen, Thomas Nordahl Petersen, Pernille Andersen, Morten Nielsen and Claus Lundegaard
# BMC Structural Biology 2009, 9:51 doi:10.1186/1472-6807-9-51
#
# Column 1: Class assignment - B for buried or E for Exposed - Threshold: 25% exposure, but not based on RSA
# Column 2: Amino acid
# Column 3: Sequence name
# Column 4: Amino acid number
# Column 5: Relative Surface Accessibility - RSA
# Column 6: Absolute Surface Accessibility
# Column 7: Z-fit score for RSA prediction
# Column 8: Probability for Alpha-Helix
# Column 9: Probability for Beta-strand
# Column 10: Probability for Coil
E D  1DQJA                  1    0.796 114.761   0.904   0.003   0.003   0.994
B I  1DQJA                  2    0.188  34.799  -0.929   0.003   0.718   0.279
E V  1DQJA                  3    0.433  66.583   1.567   0.001   0.900   0.099
B L  1DQJA                  4    0.124  22.668   0.677   0.001   0.900   0.099
E T  1DQJA                  5    0.439  60.959   0.616   0.002   0.816   0.182
B Q  1DQJA                  6    0.167  29.880   0.357   0.004   0.514   0.481
E S  1DQJA                  7    0.347  40.715   0.188   0.004   0.138   0.858
E P  1DQJA                  8    0.503  71.362  -1.020   0.018   0.019   0.964
E A  1DQJA                  9    0.555  61.150  -1.169   0.018   0.088   0.893
E T  1DQJA                 10    0.340  47.172  -1.284   0.021   0.451   0.528
B L  1DQJA                 11    0.237  43.376  -0.274   0.021   0.756   0.223
E S  1DQJA                 12    0.468  54.861  -0.632   0.018   0.846   0.136
B V  1DQJA                 13    0.191  29.326   0.055   0.023   0.655   0.322
E T  1DQJA                 14    0.396  54.995   0.217   0.004   0.420   0.576
E P  1DQJA                 15    0.529  75.008   0.109   0.004   0.085   0.910
E G  1DQJA                 16    0.663  52.194  -1.120   0.005   0.015   0.979
E D  1DQJA                 17    0.500  72.050   0.377   0.004   0.138   0.858
E S  1DQJA                 18    0.567  66.417   0.969   0.004   0.616   0.381
B V  1DQJA                 19    0.073  11.205   0.109   0.001   0.900   0.099
E S  1DQJA                 20    0.440  51.568   1.523   0.001   0.959   0.040
B L  1DQJA                 21    0.055   9.997   0.292   0.001   0.959   0.040
E S  1DQJA                 22    0.357  41.899   0.951   0.001   0.900   0.099
B C  1DQJA                 23    0.029   4.128   0.029   0.001   0.900   0.099
E R  1DQJA                 24    0.419  95.905   0.896   0.001   0.900   0.099
B A  1DQJA                 25    0.170  18.723  -0.890   0.002   0.816   0.182
E S  1DQJA                 26    0.374  43.786  -0.958   0.004   0.514   0.481
E Q  1DQJA                 27    0.596 106.517  -2.419   0.004   0.085   0.910
E S  1DQJA                 28    0.440  51.591  -2.028   0.005   0.045   0.951
E I  1DQJA                 29    0.458  84.656  -1.556   0.004   0.138   0.858
E S  1DQJA                 30    0.428  50.173  -1.662   0.004   0.138   0.858
B N  1DQJA                 31    0.271  39.645  -0.986   0.005   0.262   0.733
B N  1DQJA                 32    0.143  20.877   0.339   0.004   0.616   0.381
B L  1DQJA                 33    0.023   4.156   0.902   0.001   0.900   0.099
B H  1DQJA                 34    0.077  13.952   0.338   0.001   0.900   0.099
B W  1DQJA                 35    0.025   6.109   0.921   0.011   0.918   0.071
B Y  1DQJA                 36    0.048  10.172   0.250   0.001   0.959   0.040
B Q  1DQJA                 37    0.123  22.004   0.434   0.001   0.900   0.099
B Q  1DQJA                 38    0.148  26.415   0.608   0.004   0.616   0.381
E K  1DQJA                 39    0.388  79.894   1.482   0.004   0.138   0.858
E S  1DQJA                 40    0.612  71.703  -0.258   0.005   0.015   0.979
E H  1DQJA                 41    0.741 134.752  -2.349   0.003   0.003   0.994
E E  1DQJA                 42    0.605 105.763   0.340   0.018   0.047   0.935
E S  1DQJA                 43    0.325  38.090  -0.868   0.005   0.045   0.951
B P  1DQJA                 44    0.109  15.524  -0.532   0.004   0.197   0.799
E R  1DQJA                 45    0.334  76.394   0.175   0.004   0.616   0.381
B L  1DQJA                 46    0.145  26.586  -0.471   0.002   0.816   0.182
B L  1DQJA                 47    0.120  21.917  -0.535   0.001   0.900   0.099
B I  1DQJA                 48    0.027   5.088  -0.490   0.001   0.959   0.040
B K  1DQJA                 49    0.137  28.263  -0.160   0.001   0.959   0.040
B Y  1DQJA                 50    0.108  23.080  -1.305   0.001   0.900   0.099
B A  1DQJA                 51    0.106  11.637  -1.312   0.004   0.616   0.381
B S  1DQJA                 52    0.206  24.167  -1.292   0.004   0.197   0.799
E Q  1DQJA                 53    0.598 106.749  -1.728   0.004   0.085   0.910
E S  1DQJA                 54    0.479  56.186  -0.946   0.005   0.045   0.951
E I  1DQJA                 55    0.601 111.222  -2.321   0.005   0.015   0.979
E S  1DQJA                 56    0.736  86.306  -1.670   0.005   0.045   0.951
E G  1DQJA                 57    0.623  48.991  -1.169   0.004   0.085   0.910
B I  1DQJA                 58    0.231  42.791   0.003   0.004   0.085   0.910
E P  1DQJA                 59    0.538  76.285  -0.600   0.004   0.138   0.858
E S  1DQJA                 60    0.385  45.063  -2.010   0.005   0.336   0.660
E R  1DQJA                 61    0.302  69.089  -0.593   0.004   0.616   0.381
B F  1DQJA                 62    0.099  19.970  -1.451   0.002   0.816   0.182
E S  1DQJA                 63    0.416  48.779  -0.620   0.003   0.718   0.279
E G  1DQJA                 64    0.325  25.538  -2.100   0.004   0.616   0.381
E S  1DQJA                 65    0.454  53.185  -1.568   0.004   0.616   0.381
E G  1DQJA                 66    0.470  37.020  -1.253   0.004   0.420   0.576
E S  1DQJA                 67    0.497  58.272  -1.580   0.005   0.262   0.733
E G  1DQJA                 68    0.605  47.590  -1.319   0.004   0.138   0.858
E T  1DQJA                 69    0.402  55.771  -0.843   0.005   0.336   0.660
E D  1DQJA                 70    0.383  55.147  -0.074   0.002   0.816   0.182
B F  1DQJA                 71    0.099  19.849  -0.026   0.001   0.900   0.099
B T  1DQJA                 72    0.250  34.675   0.052   0.001   0.900   0.099
B L  1DQJA                 73    0.045   8.331   0.390   0.001   0.900   0.099
E S  1DQJA                 74    0.300  35.148   0.466   0.001   0.900   0.099
B I  1DQJA                 75    0.083  15.392  -0.121   0.002   0.816   0.182
E N  1DQJA                 76    0.400  58.501   0.207   0.004   0.616   0.381
E S  1DQJA                 77    0.439  51.474  -1.176   0.004   0.420   0.576
E V  1DQJA                 78    0.328  50.398  -0.918   0.021   0.279   0.699
E E  1DQJA                 79    0.462  80.746   0.226   0.021   0.279   0.699
E T  1DQJA                 80    0.404  56.021  -0.860   0.020   0.205   0.775
E E  1DQJA                 81    0.466  81.463  -0.463   0.021   0.279   0.699
E D  1DQJA                 82    0.499  71.978  -0.718   0.021   0.279   0.699
E F  1DQJA                 83    0.392  78.594  -0.993   0.022   0.359   0.619
B G  1DQJA                 84    0.162  12.789  -1.138   0.004   0.616   0.381
E M  1DQJA                 85    0.359  71.896   0.303   0.002   0.816   0.182
B Y  1DQJA                 86    0.055  11.668   0.973   0.001   0.900   0.099
B F  1DQJA                 87    0.142  28.540   0.774   0.001   0.900   0.099
B C  1DQJA                 88    0.039   5.518  -0.868   0.002   0.816   0.182
B Q  1DQJA                 89    0.125  22.396  -0.572   0.023   0.655   0.322
B Q  1DQJA                 90    0.148  26.361  -0.601   0.022   0.359   0.619
B S  1DQJA                 91    0.256  30.003  -0.923   0.021   0.279   0.699
B N  1DQJA                 92    0.306  44.784  -1.431   0.064   0.216   0.721
E S  1DQJA                 93    0.450  52.763  -1.632   0.056   0.142   0.802
B W  1DQJA                 94    0.304  73.112  -1.550   0.052   0.084   0.864
B P  1DQJA                 95    0.306  43.421  -1.341   0.056   0.142   0.802
E Y  1DQJA                 96    0.379  81.056  -1.213   0.052   0.084   0.864
E T  1DQJA                 97    0.395  54.800  -1.370   0.052   0.084   0.864
E F  1DQJA                 98    0.335  67.315  -1.259   0.018   0.088   0.893
E G  1DQJA                 99    0.344  27.041  -2.133   0.018   0.088   0.893
E G  1DQJA                100    0.394  30.968  -1.356   0.019   0.141   0.840
E G  1DQJA                101    0.398  31.307  -1.774   0.020   0.205   0.775
E T  1DQJA                102    0.334  46.395  -1.327   0.021   0.279   0.699
E K  1DQJA                103    0.397  81.581  -1.095   0.022   0.359   0.619
E L  1DQJA                104    0.344  63.078  -0.975   0.066   0.296   0.638
E E  1DQJA                105    0.389  67.871  -1.056   0.064   0.216   0.721
E I  1DQJA                106    0.314  58.127  -1.694   0.066   0.296   0.638
E K  1DQJA                107    0.390  80.182  -0.962   0.069   0.386   0.545
E R  1DQJA                108    0.436  99.730  -1.539   0.069   0.386   0.545
E A  1DQJA                109    0.453  49.943  -1.325   0.066   0.296   0.638
E D  1DQJA                110    0.378  54.484  -1.131   0.064   0.216   0.721
E A  1DQJA                111    0.469  51.640  -1.194   0.056   0.142   0.802
E A  1DQJA                112    0.385  42.438  -0.984   0.019   0.141   0.840
E P  1DQJA                113    0.385  54.660  -1.499   0.018   0.088   0.893
E T  1DQJA                114    0.465  64.495  -1.737   0.018   0.088   0.893
E V  1DQJA                115    0.435  66.906  -1.609   0.056   0.142   0.802
E S  1DQJA                116    0.480  56.233  -1.904   0.056   0.142   0.802
E I  1DQJA                117    0.413  76.460  -1.957   0.056   0.142   0.802
B F  1DQJA                118    0.308  61.816  -2.691   0.056   0.142   0.802
E P  1DQJA                119    0.350  49.708  -0.975   0.052   0.084   0.864
B P  1DQJA                120    0.277  39.292  -1.647   0.052   0.084   0.864
E S  1DQJA                121    0.383  44.923  -1.291   0.056   0.142   0.802
E S  1DQJA                122    0.372  43.610  -1.551   0.118   0.150   0.732
E E  1DQJA                123    0.367  64.062  -2.022   0.118   0.150   0.732
E Q  1DQJA                124    0.366  65.332  -1.929   0.118   0.150   0.732
E L  1DQJA                125    0.446  81.608  -1.310   0.113   0.087   0.800
E T  1DQJA                126    0.421  58.365  -1.418   0.053   0.043   0.903
E S  1DQJA                127    0.564  66.101  -1.604   0.018   0.047   0.935
E G  1DQJA                128    0.463  36.470  -1.384   0.018   0.088   0.893
E G  1DQJA                129    0.384  30.244  -0.314   0.005   0.336   0.660
B A  1DQJA                130    0.056   6.215  -0.820   0.004   0.616   0.381
B S  1DQJA                131    0.148  17.310   0.492   0.002   0.816   0.182
B V  1DQJA                132    0.030   4.688   0.393   0.001   0.959   0.040
B V  1DQJA                133    0.053   8.161  -0.144   0.001   0.959   0.040
B C  1DQJA                134    0.018   2.513   0.724   0.001   0.900   0.099
B F  1DQJA                135    0.132  26.512  -0.333   0.001   0.900   0.099
B L  1DQJA                136    0.053   9.631  -1.035   0.004   0.616   0.381
B N  1DQJA                137    0.242  35.473  -0.991   0.005   0.262   0.733
E N  1DQJA                138    0.417  61.005  -2.840   0.004   0.138   0.858
B F  1DQJA                139    0.271  54.370  -2.672   0.004   0.085   0.910
B Y  1DQJA                140    0.196  41.907  -1.167   0.004   0.085   0.910
B P  1DQJA                141    0.294  41.676  -1.827   0.004   0.138   0.858
E K  1DQJA                142    0.410  84.337  -2.199   0.004   0.197   0.799
E D  1DQJA                143    0.319  45.953  -0.861   0.005   0.336   0.660
B I  1DQJA                144    0.054   9.971  -0.322   0.004   0.514   0.481
B N  1DQJA                145    0.192  28.036  -0.449   0.002   0.816   0.182
B V  1DQJA                146    0.044   6.824  -0.710   0.001   0.900   0.099
B K  1DQJA                147    0.172  35.319  -0.382   0.001   0.900   0.099
B W  1DQJA                148    0.070  16.859  -0.813   0.011   0.918   0.071
B K  1DQJA                149    0.231  47.414  -0.111   0.002   0.816   0.182
E I  1DQJA                150    0.391  72.409   0.305   0.004   0.514   0.481
E D  1DQJA                151    0.665  95.769  -0.475   0.005   0.045   0.951
E G  1DQJA                152    0.738  58.073  -2.251   0.005   0.015   0.979
E S  1DQJA                153    0.686  80.352  -0.555   0.004   0.197   0.799
E E  1DQJA                154    0.528  92.329  -1.141   0.004   0.138   0.858
E R  1DQJA                155    0.287  65.654  -0.627   0.018   0.088   0.893
E Q  1DQJA                156    0.489  87.353  -1.230   0.020   0.205   0.775
E N  1DQJA                157    0.386  56.584  -1.378   0.022   0.359   0.619
B G  1DQJA                158    0.300  23.634  -1.919   0.022   0.552   0.426
B V  1DQJA                159    0.255  39.132  -0.974   0.018   0.846   0.136
B L  1DQJA                160    0.233  42.626  -0.613   0.018   0.846   0.136
E N  1DQJA                161    0.410  59.965  -0.951   0.018   0.846   0.136
B S  1DQJA                162    0.260  30.495  -1.400   0.022   0.552   0.426
E W  1DQJA                163    0.358  86.075  -0.402   0.022   0.552   0.426
E T  1DQJA                164    0.457  63.386  -0.913   0.022   0.359   0.619
E D  1DQJA                165    0.382  55.046  -0.946   0.020   0.205   0.775
E Q  1DQJA                166    0.564 100.713  -1.744   0.020   0.205   0.775
E D  1DQJA                167    0.587  84.558  -2.129   0.018   0.088   0.893
E S  1DQJA                168    0.588  68.914  -1.526   0.018   0.047   0.935
E K  1DQJA                169    0.527 108.507  -0.738   0.018   0.019   0.964
E D  1DQJA                170    0.694 100.020  -1.542   0.005   0.015   0.979
E S  1DQJA                171    0.511  59.831  -1.897   0.005   0.045   0.951
E T  1DQJA                172    0.342  47.380   0.078   0.004   0.420   0.576
B Y  1DQJA                173    0.080  17.181  -1.056   0.003   0.718   0.279
B S  1DQJA                174    0.109  12.798  -1.121   0.002   0.816   0.182
B M  1DQJA                175    0.064  12.826  -1.574   0.001   0.900   0.099
B S  1DQJA                176    0.151  17.756  -0.547   0.001   0.959   0.040
B S  1DQJA                177    0.044   5.169  -1.858   0.001   0.959   0.040
B T  1DQJA                178    0.213  29.571   0.038   0.001   0.900   0.099
B L  1DQJA                179    0.045   8.221  -0.434   0.001   0.900   0.099
E T  1DQJA                180    0.323  44.814   0.712   0.001   0.900   0.099
B L  1DQJA                181    0.077  14.044   0.445   0.002   0.816   0.182
E T  1DQJA                182    0.402  55.688   1.137   0.021   0.451   0.528
E K  1DQJA                183    0.472  97.173  -0.839   0.021   0.279   0.699
E D  1DQJA                184    0.535  77.122  -0.836   0.021   0.279   0.699
E E  1DQJA                185    0.525  91.805   0.683   0.021   0.279   0.699
B Y  1DQJA                186    0.225  48.147  -0.687   0.019   0.141   0.840
E E  1DQJA                187    0.674 117.730   0.249   0.005   0.045   0.951
E R  1DQJA                188    0.614 140.560   0.681   0.005   0.045   0.951
E H  1DQJA                189    0.618 112.414  -0.346   0.005   0.045   0.951
E N  1DQJA                190    0.464  67.944   0.009   0.005   0.336   0.660
E S  1DQJA                191    0.430  50.349   1.610   0.002   0.816   0.182
B Y  1DQJA                192    0.059  12.694   1.120   0.001   0.959   0.040
B T  1DQJA                193    0.273  37.907   1.840   0.001   0.959   0.040
B C  1DQJA                194    0.022   3.019   0.947   0.001   0.959   0.040
B E  1DQJA                195    0.204  35.639   1.235   0.001   0.959   0.040
B A  1DQJA                196    0.073   8.100  -0.110   0.001   0.959   0.040
E T  1DQJA                197    0.388  53.802   0.621   0.001   0.959   0.040
B H  1DQJA                198    0.132  23.974   0.356   0.002   0.816   0.182
E K  1DQJA                199    0.619 127.390  -0.105   0.005   0.262   0.733
E T  1DQJA                200    0.632  87.617  -1.960   0.005   0.045   0.951
E S  1DQJA                201    0.444  52.060  -0.708   0.005   0.045   0.951
E T  1DQJA                202    0.606  84.066  -0.070   0.018   0.088   0.893
E S  1DQJA                203    0.573  67.202   0.653   0.020   0.205   0.775
E P  1DQJA                204    0.393  55.724   0.347   0.022   0.359   0.619
B I  1DQJA                205    0.142  26.251  -0.248   0.003   0.718   0.279
E V  1DQJA                206    0.537  82.598   0.524   0.001   0.900   0.099
E K  1DQJA                207    0.318  65.413   1.492   0.001   0.900   0.099
E S  1DQJA                208    0.488  57.158   0.247   0.001   0.900   0.099
B F  1DQJA                209    0.090  18.103   0.467   0.003   0.718   0.279
E N  1DQJA                210    0.525  76.933   1.872   0.004   0.514   0.481
E R  1DQJA                211    0.462 105.821   0.716   0.019   0.141   0.840
E N  1DQJA                212    0.660  96.565  -0.840   0.018   0.088   0.893
E E  1DQJA                213    0.650 113.468   1.628   0.018   0.088   0.893
E C  1DQJA                214    0.450  63.138  -1.206   0.003   0.003   0.994

Explain the output.  Go back.
```

---
